# Analysis of MicroRNA Expression Profiles in Weaned Pig Skeletal Muscle after Lipopolysaccharide Challenge

**DOI:** 10.3390/ijms160922438

**Published:** 2015-09-16

**Authors:** Jing Zhang, Shu-Lin Fu, Yan Liu, Yu-Lan Liu, Wen-Jun Wang

**Affiliations:** 1 Hubei Collaborative Innovation Center for Animal Nutrition and Feed Safety, Hubei Key Laboratory of Animal Nutrition and Feed Science, Wuhan Polytechnic University, Wuhan 430023, China; E-Mails: judyzhang1103@126.com (J.Z.); sharonliuyan@126.com (Y.L.); 2 Wuhan Institute of Animal Husbandry and Veterinary Science, Wuhan Academy of Agricultural Science & Technology, Wuhan 430208, China; E-Mail: fushulin@webmail.hzau.edu.cn; 3 College of Life Sciences, South-Central University for Nationalities, Wuhan 430074, China

**Keywords:** skeletal muscle wasting, LPS, microRNA, small RNA sequencing, pig

## Abstract

MicroRNAs (miRNAs) constitute a class of non-coding RNAs that play a crucial regulatory role in skeletal muscle development and disease. Several acute inflammation conditions including sepsis and cancer are characterized by a loss of skeletal muscle due primarily to excessive muscle catabolism. As a well-known inducer of acute inflammation, a lipopolysaccharide (LPS) challenge can cause serious skeletal muscle wasting. However, knowledge of the role of miRNAs in the course of inflammatory muscle catabolism is still very limited. In this study, RNA extracted from the skeletal muscle of pigs injected with LPS or saline was subjected to small RNA deep sequencing. We identified 304 conserved and 114 novel candidate miRNAs in the pig. Of these, four were significantly increased in the LPS-challenged samples and five were decreased. The expression of five miRNAs (ssc-miR-146a-5p, ssc-miR-221-5p, ssc-miR-148b-3p, ssc-miR-215 and ssc-miR-192) were selected for validation by quantitative polymerase chain reaction (qPCR), which found that ssc-miR-146a-5p and ssc-miR-221-5p were significantly upregulated in LPS-challenged pig skeletal muscle. Moreover, we treated mouse C2C12 myotubes with 1000 ng/mL LPS as an acute inflammation cell model. Expression of TNF-α, IL-6, muscle atrophy F-box (MAFbx) and muscle RING finger 1 (MuRF1) mRNA was strongly induced by LPS. Importantly, miR-146a-5p and miR-221-5p also showed markedly increased expression in LPS-treated C2C12 myotubes, suggesting the two miRNAs may be involved in muscle catabolism systems in response to acute inflammation caused by a LPS challenge. To our knowledge, this study is the first to examine miRNA expression profiles in weaned pig skeletal muscle challenged with LPS, and furthers our understanding of miRNA function in the regulation of inflammatory muscle catabolism.

## 1. Introduction

Skeletal muscle is the most abundant tissue in the body and its main functions include supporting body structure, controlling motor movements and storing energy. As a major site of metabolic activity, skeletal muscle is considered the natural reserve of amino acids and energy for times of need [1]. Thus, a minor change in muscle protein metabolism has a major impact on whole body metabolism. The balance between protein synthesis and degradation governs muscle mass and function. Severe infection and sepsis can cause rapid loss of muscle mass and myofibrillar proteins (muscle atrophy/wasting) [2].

Lipopolysaccharide (LPS), the outer membrane of Gram-negative bacteria, is a potent activator of the innate immune system through recognition by Toll-like receptor 4 (TLR4) [3,4]. LPS significantly reduced protein synthesis in longissimus dorsi muscle by 11% and in gastrocnemius by 15% in newborn piglets [5]. Similarly, *in vitro* LPS led to a significant 50% decrease in protein synthesis in mouse myoblast C2C12 cells [6]. Complex mechanisms contribute to LPS-induced muscle wasting. LPS activates several signaling pathways by acting on TLR4 to elevate inflammatory gene expression. Proinflammatory cytokines have been reported to promote muscle protein degradation by upregulating expression of two muscle-specific E3 ubiquitin ligases, MAFbx and MuRF1 [7,8]. However, activation of p38 MAPK and NF-κB pathways by LPS can upregulate MAFbx and MuRF1 directly, independent of humoral factors [9,10]. A recent study found that TLR4 mediates LPS-induced muscle wasting via coordinated activation of ubiquitin-proteasome and autophagy-lysosome pathways [11]. Considering the complexity of the muscle atrophic program, other levels of regulation may also be involved.

MicroRNAs (miRNAs) represent a class of approximately 22 nucleotide (nt) non-coding RNAs that post-transcriptionally regulate gene expression by translational repression or degradation of transcripts. Previous studies have demonstrated that miRNAs are involved in skeletal muscle development, fiber-type switch and regeneration [12,13]. Aberrant regulation of some muscle-enriched miRNAs (referred to as myomiRs) is associated with some pathological conditions [14,15]. Recent studies have shown that miRNAs play a key regulatory role in skeletal muscle atrophy. For example, inhibition of miRNA-206 in SOD1^G93A^ transgenic mice induced severe atrophy [16]. Activation of the forkhead box O3 (FoxO3) transcription factor also caused skeletal muscle atrophy, and miR-182 attenuated atrophy-related gene expression by targeting FoxO3 in skeletal muscle [17]. miR-23a was recently shown to decrease MAFbx and MuRF1 expression in skeletal muscle [18].

Many studies have demonstrated that miRNA expression profiles are subject to change in different cells when stimulated by LPS via TLR-signaling pathways, including miR-146a, miR-155, miR-132, miR-15a/16, miR-27a and miR-532-5p [19,20,21,22,23]. Moreover, miRNAs regulate TLR-signaling pathways by targeting multiple molecules [24]. These results suggest a potential role for miRNAs in LPS-induced inflammatory muscle wasting. However, until now, the involvement of miRNAs in the course of inflammatory muscle wasting has been largely unknown. In this study, we performed high-throughput sequencing and used bioinformatics tools to obtain expression profiles of miRNAs in weaned pig skeletal muscle after a LPS or saline challenge. Conserved and novel miRNAs were identified and differentially-expressed miRNAs were analyzed. The miRNA information obtained from our study will contribute to the further elucidation of miRNA function in the regulation of inflammatory muscle catabolism.

## 2. Results and Discussion

### 2.1. General Analysis of Small RNAs

In order to investigate the role that miRNAs play in inflammatory muscle catabolism, we established a weaned piglet model of LPS-induced acute inflammation. Groups of pigs (*n* = 6) received an i.p. injection of either LPS or saline. Four hours after administration, we collected blood and gastrocnemius muscle samples from individual animals.

**Figure 1:**
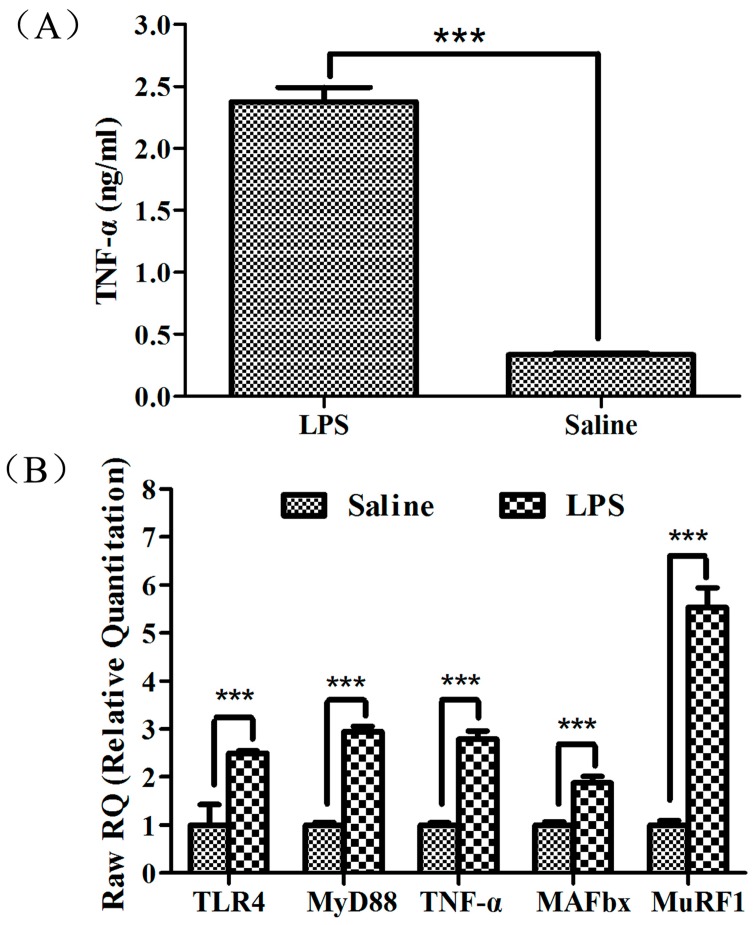
Lipopolysaccharide (LPS)-induced acute inflammation and muscle protein degradation. (**A**) Plasma TNF-α concentrations in weanling pigs were measured 4 h after treatment with LPS or saline; and (**B**) TLR4, MyD88, TNF-α, MAFbx and MuRF1 mRNA expression were determined by qPCR in the skeletal muscles of weanling pigs 4 h after treatment with LPS or saline. *** *p* < 0.001 *vs*. saline control. TLR4: Toll-like receptor 4.

As a potent agonist of TLR4, LPS can activate TLR4 signaling, which in turn induces the expression of the inflammatory cytokines TNF-α, IL-1 and IL-6 [25]. In this study, pigs injected with LPS had higher plasma TNF-α concentrations (Figure 1A; *p* < 0.001), indicating acute inflammation was induced by the LPS challenge. Associated with muscle atrophy and protein degradation is a rapid and sustained increase in MuRF1 and MAFbx expression, two muscle-specific ubiquitin ligase E3 proteins thought to target specific proteins for degradation by the 26S proteasome [26]. Systemically elevated inflammatory cytokines are thought to mediate muscle wasting by upregulating MAFbx and MuRF1 [27]. In our study, MAFbx, MuRF1, TNF-α, TLR4 and MyD88 in gastrocnemius muscle were all significantly higher in pigs treated with LPS than in those treated with saline (Figure 1B; *p* < 0.001), suggesting that LPS induced muscle protein degradation in our piglet model. Skeletal muscle samples were then used for small RNA sequencing.

**Figure 2:**
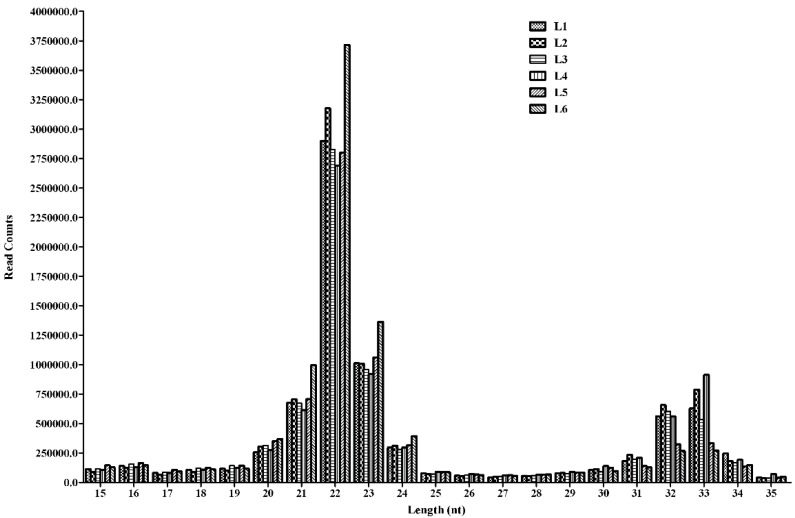
Length distribution of sequencing reads in the six libraries.

**Figure 3:**
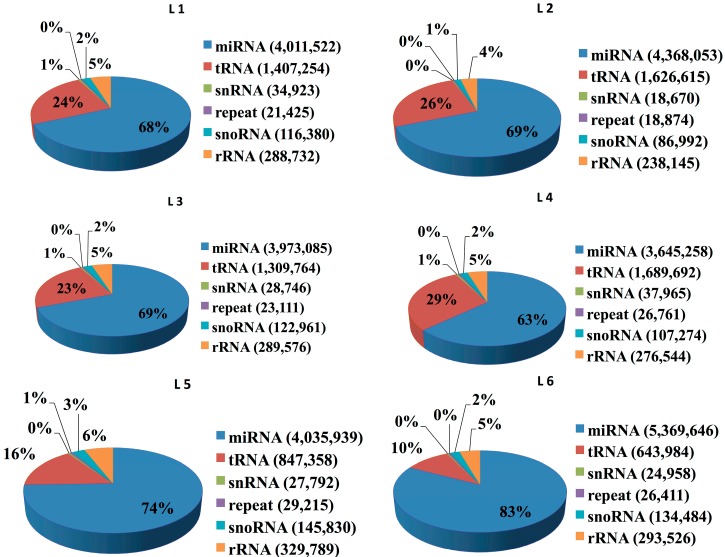
Distribution of total sequenced reads among various RNA classes in the six libraries.

Three independent RNA libraries were constructed for the LPS and saline groups and then deep sequenced using the Illumina Hiseq2500 system. A pool of two samples from the LPS and saline injected piglets was used to create each RNA library. The six small RNA libraries from LPS groups L1–L3 and saline groups L4–L6 yielded a total of 10,207,581, 9,899,333, 10,329,995, 10,175,216, 9,729,242 and 10,385,051 raw reads, respectively. After eliminating adaptor and low-quality reads, a total of 8,166,036, 8,578,151, 7,974,032, 8,261,735, 7,740,201 and 9,095,380 clean reads were obtained in the L1–L6 libraries, respectively (Table 1). All clean reads were then aligned to the pig genome databases, miRBase, Rfam, RepBase and mRNA/EST (Table 1). The sequence length distribution in the six libraries exhibited wide variation ranging from 15 to 35 nt. Most of the small RNAs were 21–23 nt in length, predominantly 22 nt, which is the typical length of Dicer-derived products (Figure 2). To assess the efficiency of deep sequencing for the detection of miRNAs, all sequence reads were annotated as belonging to different categories of RNA (rRNA, snRNA, siRNA, snoRNA, tRNA, miRNA) by performing a BLASTN search against the Rfam and miRBase databases. The total rRNA proportion is regarded as a marker of sample quality and generally should be less than 40% in animal samples of high quality. Our results showed that most of the annotated small RNAs were miRNAs comprising 63%–83% of the total clean reads in the six libraries, suggesting that the skeletal muscle samples used in this study were of high quality (Figure 3).

**Table 1:** Preliminary analysis of high-throughput sequencing data.

Classes of Reads	The LPS-Challenged Group						The Saline-Treated Group					
	L1 Library		L2 Library		L3 Library		L4 Library		L5 Library		L6 Library	
	Total	Unique	Total	Unique	Total	Unique	Total	Unique	Total	Unique	Total	Unique
Clean date	8,166,036	424,320	8,578,151	393,231	7,974,032	426,707	8,261,735	532,894	7,740,201	456,559	9,095,380	536,545
Genome	7,392,469	306,807	7,875,613	288,075	7,280,396	316,514	7,339,331	383,868	6,784,012	329,527	7,966,211	395,908
miRBase (mature)	3,979,370	7498	4,339,317	7753	3,940,011	7770	3,609,976	7597	3,995,522	8251	5,332,846	8842
Rfam	1,972,825	71,199	2,119,590	65,099	1,847,538	74,226	2,269,168	81,568	1,466,801	85,054	1,304,709	86,567
RepBase	166,565	28,787	132,226	25,094	157,402	30,124	259,354	31,758	180,018	30,089	158,490	35,920
mRNA/EST	501,200	128,735	437,848	116,045	516,196	129,355	608,415	173,253	594,752	126,662	586,024	183,958

L1, L2 and L3 represent three independent RNA libraries for LPS groups; L4, L5 and L6 represent three independent RNA libraries for saline groups. LPS: lipopolysaccharide.

### 2.2. Identification of Known miRNAs

By performing BLASTN searches against the miRBase (version 21.0), a total of 304 known miRNAs corresponding to 411 mature miRNAs were identified (Table S1). The reads of these miRNAs ranged from 1 to 1,515,145, indicating that not only highly-expressed miRNAs, but also weakly-expressed miRNAs were identified by the Illumina Hiseq2500 system.

**Figure 4:**
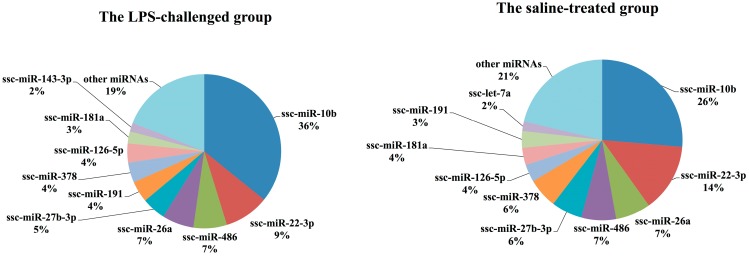
Top 10 most abundant miRNAs in the LPS-challenged and saline-treated groups, ordered by the average proportion of each miRNA read relative to total normalized miRNA reads.

We considered the relative number of normalized sequence reads of each miRNA as an indication of its abundance in each sample. The top ten most abundant miRNAs in the two groups were ordered by the average proportion of each miRNA read relative to the total normalized miRNA reads (Figure 4). The top nine most abundant miRNAs shared between the two groups were ssc-miR-10b, ssc-miR-22-3p, ssc-miR-486, ssc-miR-26a, ssc-miR-27b-3p, ssc-miR-191, ssc-miR-378, ssc-126-5p and ssc-miR-181. Previous studies have demonstrated that the myomiRs miR-1, miR-133a/b, miR-206, miR-486, miR-26a, miR-27b, miR-378, miR-148a and miR-181 are highly enriched in skeletal muscle and play a key role in skeletal muscle metabolism [28,29,30,31]. In our sequencing libraries, five of these known myomiRs (miR-486, miR-26a, miR-27b, miR-378 and miR-181) were identified with the greatest abundance, accounting for 26% and 29% of the total normalized miRNA reads in the LPS-challenged and saline-treated groups, respectively. In contrast, miR-1, miR-133a/b and miR-206, as muscle-specific miRNAs, were not the most abundant miRNAs in our data. This observation is not in agreement with other studies of miRNAs in pig skeletal muscle [32,33]; instead, miR-10b had the highest expression in both groups of our study, accounting for 36% and 26% of total normalized miRNA reads, respectively. MiR-191 has been reported to regulate important cellular processes such as cell proliferation, differentiation, apoptosis and migration [34]. miR-126-5p is an intronic miRNA identified as a tumor suppressor in many tumors [35]. There is very little specific information on the expression and function of miR-126-5p and miR-191 in skeletal muscle, yet these two miRNAs were identified as two of the more abundant miRNAs in our data.

### 2.3. Identification of Novel miRNAs by miRDeep2

**Figure 5:**
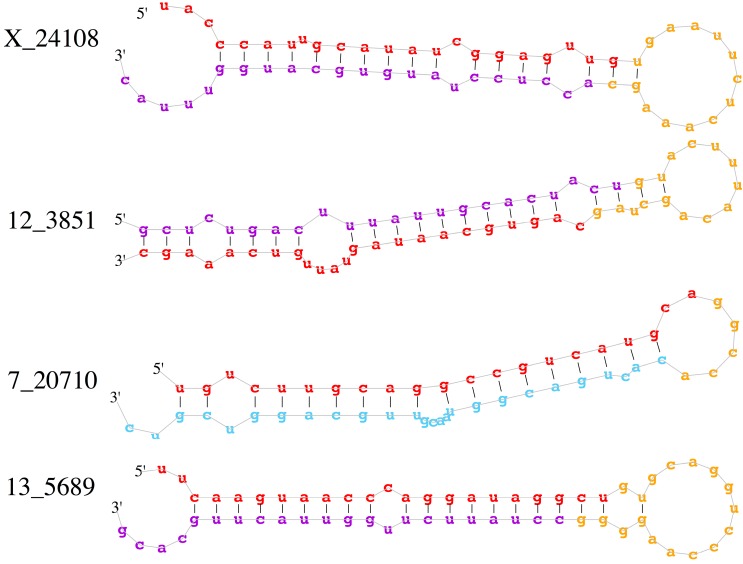
Predicted stem-loop secondary structures of four candidate novel pig miRNAs. Red: mature sequence; purple: star sequence with no reads cover; blue: star sequence with no reads covered; yellow: stem sequence.

To identify novel miRNAs in our deep sequencing data, the clean miRNA reads were aligned to the pig genome and analyzed by miRDeep2 (version 2.0.05), an algorithm based on the miRNA biogenesis model [36]. miRDeep2 aligns mapped reads to potential miRNA-like hairpin structures and assigns scores to measure the probability that hairpins are true miRNA precursors. miRDeep2 detected 259 known miRNAs and predicted 156 potential novel miRNAs at the relatively stringent score cut-off of 4 and signal-to-noise ratio of 17.3 (Table 2). The predicted novel miRNAs were then filtered by removal of loci matching other RNA genes or genomic repeats, which reduced the list to 114 candidate novel miRNAs with significant randfold *p*-values (*p* < 0.05) (Table S2). The expression levels of the novel miRNAs were relatively low. Among these 114 new miRNAs, only 10 miRNAs were sequenced more than 100 times, including X_24108, 12_3851, 7_20710, 13_5689, X_24098, X_24105, 14_7436, 5_17689, X_24105 and 3_13965 (Table S3). Secondary structures for some candidates (X_24108, 12_3851, 7_20710 and 13_5689) are shown in Figure 5.

**Table 2:** Survey by miRDeep2 analysis showing the number of novel and known miRNAs under different score cut-offs ranging from 10 to 1.

miRDeep2 Score ^1^	Novel miRNAs			Known miRNAs			-
	Predicted ^2^	False Positives ^3^	True Positives ^4^	In Species ^5^	In Data ^6^	Detected ^7^	Signal-to-Noise ^8^
10	65	3 ± 1	62 ± 1 (96% ± 2%)	411	304	222 (72%)	43.7
9	68	3 ± 1	65 ± 1 (96% ± 2%)	411	304	223 (73%)	43.5
8	69	3 ± 2	66 ± 3 (96% ± 2%)	411	304	223 (73%)	42.7
7	73	3 ± 2	70 ± 2 (96% ± 2%)	411	304	223 (73%)	42
6	78	3 ± 2	75 ± 3 (96% ± 2%)	411	304	223 (73%)	39.7
5	126	6 ± 2	120 ± 3 (95% ± 2%)	411	304	256 (83%)	30.7
4	156	15 ± 4	141 ± 4 (90% ± 3%)	411	304	259 (84%)	17.3
3	170	42 ± 6	128 ± 5 (75% ± 4%)	411	304	261 (85%)	7.5
2	206	57 ± 7	149 ± 6 (72% ± 3%)	411	304	265 (86%)	6.2
1	300	81 ± 8	219 ± 7 (73% ± 3%)	411	304	278 (91%)	5.4

^1^ The miRDeep2 score represents the log-odds probability of a sequence being a genuine miRNA precursor *versus* the probability that it is a background hairpin, given the evidence from the data; ^2^ Number of novel miRNA hairpins with a score ≥cut-off; ^3^ Number of false positive miRNA hairpins predicted at this cut-off, as estimated by the miRDeep2 controls. Mean and standard deviation are estimated from 100 rounds of permuted controls; ^4^ Number of true positive miRNA hairpins is estimated as *t* = total novel miRNAs—false positive novel miRNAs. The percentage of the predicted novel miRNAs that are estimated to be true positives is calculated as *p* = *t*/total novel miRNAs. The number of false positives is estimated from 100 rounds of permuted controls. In each of the 100 rounds, *t* and *p* are calculated, generating means and standard deviations of *t* and *p*. The variable *p* can be used as an estimation of the miRDeep2 positive predictive value at the score cut-off; ^5^ Number of reference mature miRNAs for the pig species given as input to miRDeep2; ^6^ Number of reference mature miRNAs that map perfectly to one or more of precursor candidates that have been excised from the genome by miRDeep2; ^7^ Number of reference mature miRNAs that map perfectly to one or more of predicted miRNA hairpins that have a score equal to or exceeding the cut-off. The percentage of reference mature miRNAs in the data that is detected by miRDeep2 is calculated as *s* = reference mature miRNAs detected/reference mature miRNAs in the data and can be used as an estimation of miRDeep2 sensitivity at the score cut-off; ^8^ For the given score cut-off, the signal-to-noise ratio is estimated as *r* = total miRNA hairpins reported/mean estimated false positive miRNA hairpins over 100 rounds of permuted controls.

### 2.4. Differential Expression Analysis

**Figure 6:**
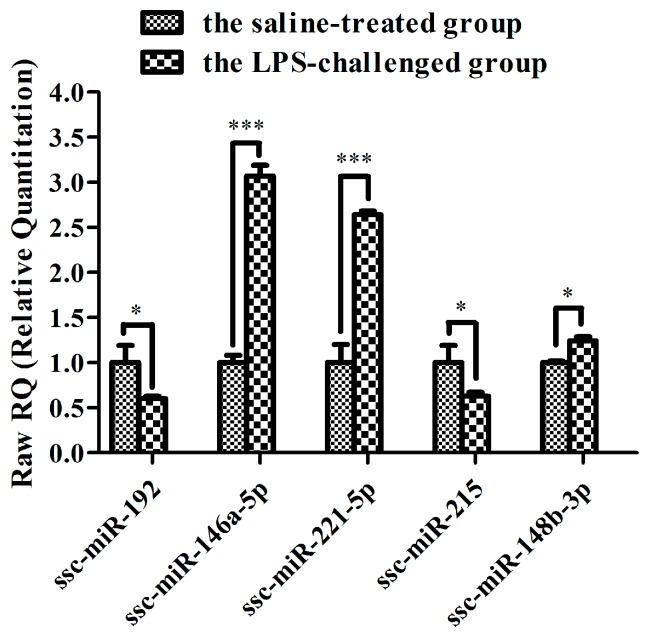
qPCR analysis of five selected miRNAs. qPCR was performed to verify expression of the five miRNAs in the same RNA samples used for RNA sequencing. Expression was normalized by U6. * *p* < 0.05, *** *p* < 0.001 *vs*. saline control.

To investigate the dynamic changes in miRNA expression in piglet skeletal muscle after an LPS challenge, we analyzed differential expression of all identified miRNAs between the LPS and saline groups. After normalization of the raw reads we found that four miRNAs (ssc-miR-146a-5p, ssc-miR-221-5p, ssc-miR-9860-5p and ssc-miR-148b-3p) were significantly upregulated in the LPS-challenged samples with a *p*-value cutoff of 0.05. Similarly, five miRNAs (ssc-miR-192, 4_16129, 13_5595, ssc-miR-215 and ssc-miR-429) were significantly downregulated (Table 3). Differential expressions of five selected miRNAs (ssc-miR-146a-5p, ssc-miR-221-5p, ssc-miR-148b-3p, ssc-miR-215 and ssc-miR-192) were validated by quantitative polymerase chain reaction (qPCR). As shown in Figure 6, qualitative qPCR validated that ssc-miR-146a-5p, ssc-miR-221-5p and ssc-miR-148b-3p were significantly upregulated by LPS, and ssc-miR-215 and ssc-miR-192 were downregulated. The qualitative qPCR results for these five miRNAs were consistent with the data from RNA-sequencing. Of note, injection of LPS resulted in significantly enhanced expression of ssc-miR-146a-5p and ssc-miR-221-5p.

**Table 3:** miRNAs significantly up- or downregulated in LPS-challenged piglet skeletal muscle.

miRNAs ID	The LPS-Challenged Group			The Saline-Treated Group			FoldChange_Log2	*p*-Value
	L1_TPM	L2_TPM	L3_TPM	L4_TPM	L5_TPM	L6_TPM		
ssc-miR-221-5p	33.69	64.15	41.10	10.94	6.95	6.59	2.50	1.65 × 10^−11^
ssc-miR-146a-5p	2.73	14.64	50.54	2.98	13.26	4.71	1.70	1.97 × 10^−5^
ssc-miR-9860-5p	8.50	9.58	12.18	3.65	4.10	3.53	1.42	1.22 × 10^−3^
ssc-miR-148b-3p	216.10	294.41	242.05	106.78	119.34	146.86	1.01	1.73 × 10^−5^
ssc-miR-192	191.82	104.61	123.61	545.85	142.38	183.34	−1.05	1.00 × 10^−2^
4_16129	9.41	3.46	7.92	27.19	13.58	9.41	−1.27	4.33 × 10^−2^
13_5595	5.77	5.86	7.92	18.57	26.20	8.94	−1.46	1.87 × 10^−2^
ssc-miR-215	16.09	1.06	1.83	43.11	4.74	18.12	−1.80	9.43 × 10^−4^
ssc-miR-429	0.61	0.00	0.61	3.32	2.53	0.94	−2.48	1.20 × 10^−2^

### 2.5. LPS Induces miR-146a-5p and miR-221-5p Expression in C2C12 Myotubes

**Figure 7:**
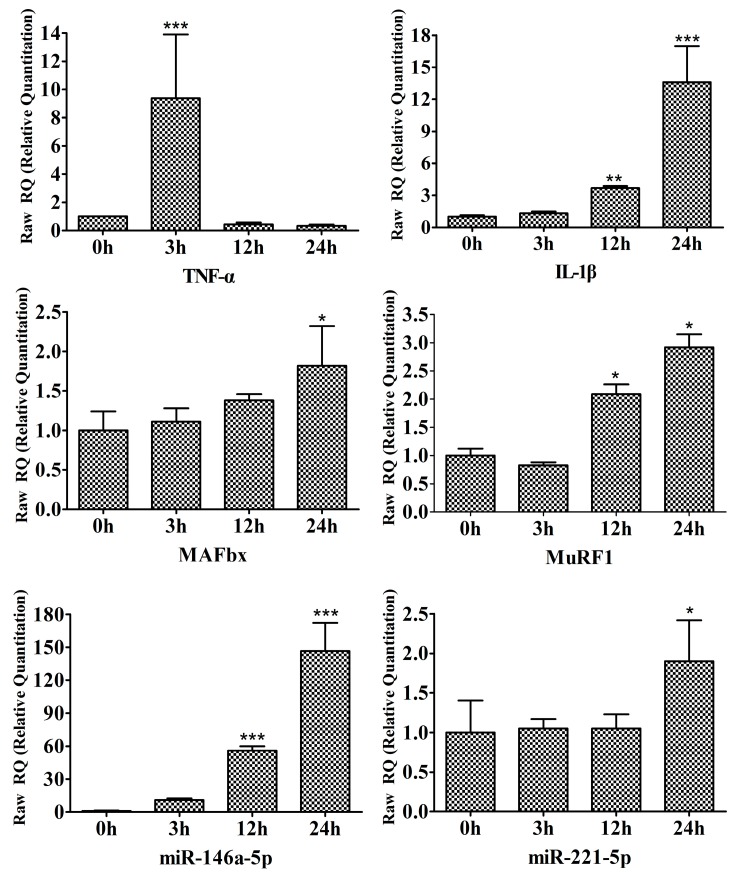
LPS upregulates the expression of miR-146a-5p and miR-221-5p in C2C12 myotubes. C2C12 myotubes were treated with 1000 ng/mL LPS for the indicated periods. Triplicate samples of cells were collected at each time point. RT-qPCR was performed to determine the time-dependent effects of LPS on the expression of TNF-α, IL-1β, MAFbx, MuRF1, miR-146a-5p and miR-221-5p. * *p* < 0.05, ** *p* < 0.01, *** *p* < 0.001 *vs*. 0 h.

In our studies, we also carried out *in vitro* evaluation of the response to LPS by C2C12 myotubes that are known to express TLR4 [3]. C2C12 myotubes were incubated with 1000 ng/mL LPS for the indicated periods (0, 3, 12 and 24 h). As shown in Figure 7, LPS treatment rapidly upregulated TNF-α and IL-1β mRNA levels at 3 h. Moreover, qPCR analysis confirmed a similar increase in MAFbx and MuRF1 mRNA in response to LPS treatment (Figure 7). These results are in agreement with other studies of LPS-induced myotube atrophy [11,37]. Interestingly, LPS treatment rapidly stimulated miR-146a-5p expression by approximately 11-fold at 3 h, which peaked in 24 h at approximately 147-fold (Figure 7). In contrast, miR-221-5p was not upregulated until 24 h of LPS treatment (Figure 7).

Accumulating evidence collectively informs us that miR-146a is a key regulator of the innate immune response [38]. miR-146a was previously found to be transcriptionally induced by NF-κB in response to activation of innate immune signaling in monocytes [21]. miR-146a has been shown both *in vitro* and *in vivo* to directly target interleukin-1 receptor-associated kinase 1 (IRAK1) and tumor necrosis factor (TNF) receptor-associated factor 6 (TRAF6), suggesting it can control cytokine and TLR signaling through a negative feedback regulatory loop [39,40,41]. Studies have also revealed a role for miR-146a in endotoxin-induced tolerance. Experiments with THP-1 macrophages demonstrated that induction of endotoxin tolerance required miR-146a upregulation and that transfection of exogenous miR-146a was sufficient to induce tolerance, even in the absence of LPS priming [42]. Conversely, inhibition of miR-146a diminished the effects of LPS tolerance [43]. Our results are consistent with previous studies that LPS induced the expression of miR-146a in piglet skeletal muscle and C2C12 myotubes in response to TLR4 signaling. We speculated that LPS-induced miR-146a-5p might act as a negative regulator of inflammatory muscle catabolism through negative feedback regulation of TLR signaling. Therefore, we analyzed the expression of IRAK1 and TRAF6 in the LPS-treated C2C12 myotubes. However, LPS induced the expressions of IRAK1 and TRAF6 at 12 h (Figure S1), suggesting miR-146a might be involved in inflammatory muscle catabolism by regulating other target genes. Therefore, further studies have to be carried out *in vitro* to identify miR-146a potential function and its target genes in inflammatory muscle catabolism process.

miR-221 is a well-known oncogenic miRNA, which is involved in tumor development by regulating cell proliferation and contributes to TNF-related apoptosis-inducing ligand (TRAIL) resistance [44,45]. However, a recent study found that miRNA-221 also regulates endothelial nitric oxide production and the inflammatory response by targeting adiponectin receptor 1 (ADIPOR1) [46]. Moreover, inflammation significantly induced miR-221 expression in adipocytes [47]. In our study, we observed greater miR-221-5p expression in LPS-injected pig skeletal muscle and LPS-treated C2C12 myotubes, suggesting that this miRNA may play a functional role in inflammatory muscle catabolism by regulating gene expression. We also analyzed the expression of ADIPOR1 in the LPS-treated C2C12. The qPCR result showed that LPS had no effect on the mRNA expression of ADIOPR1 (data not shown). However, microRNA regulate gene expression by translational repression or degradation of transcripts. Thus, further investigation would be focused on the potential function manners of miR-221-5p, targets identification and knock-out experiment at celluar level.

## 3. Experimental Section

### 3.1. Animal and Tissue Collection

A total of 12 weaned barrows (Duroc × Large White × Landrace; weaned at 21 ± 1 day of age; 8.87 ± 0.72 kg body weight) were randomly allotted to two groups. The pigs in the experimental group (*n* = 6) were injected i.p. with LPS (*Escherichia coli* serotype 055:B5, Sigma Chemical, St. Louis, MO, USA) at 100 µg/kg body weight (BW). The control group (*n* = 6) was injected with the same volume of saline solution. After 4 h treatment with LPS or saline, blood samples from these 12 pigs were collected from the anterior vena cava via venipuncture to obtain plasma. The pigs were then sacrificed according to the protocols set forth by the HuBei Province, China Biological Studies Animal Care and Use Committee. The gastrocnemius muscles were dissected and snap-frozen in liquid nitrogen.

### 3.2. TNF-α Concentration in Plasma Measurements

Plasma IL-6 and TNF-α were measured using commercially available porcine ELISA kits (R&D Systems, Inc., Minneapolis, MN, USA) as previously described [48].

### 3.3. Reverse Transcription qPCR

miRNA expression was analyzed using a Hairpin-it™ miRNA qPCR Quantitation Kit (GenePharma, Shanghai, China) and the Applied Biosystems 7500 Real-Time PCR system (Applied Biosystems, Foster City, CA, USA), according to the manufacturer. U6 was used as a normalization gene, and all data were analyzed using the 2^−∆∆*C*t^ method [49].

Expression of TNF-α, TLR4, MyD88, MAFbx and MuRF1 mRNA was analyzed using the Applied Biosystems 7500 Real-Time PCR system. cDNA synthesis and reverse transcription qPCR (RT-qPCR) were carried out as previously descried [48]. Glyceraldehyde 3-phosphate dehydrogenase (GAPDH) was used as an internal normalization control. Sequences of specific primers are shown in Table S4. All data were analyzed using the 2^−∆∆*C*t^ method [49].

### 3.4. Small-RNA Library Construction and Deep Sequencing

Total RNA was isolated from skeletal muscle samples using Trizol (Invitrogen, Carlsbad, CA, USA) according to the manufacturer’s protocol. For each small RNA library construction, 1 μg of total RNA from each sample was used as starting material. Three experimental small RNA libraries and three control small RNA libraries were constructed in order to count with biological replicates. Two samples from LPS-challenged piglets were pooled to create each experimental library and each control small RNA library was constructed out of two pooled samples from saline-treated piglets. The pooled RNAs were ligated to a 5′ and a 3′ adapter sequentially, followed by reverse transcription and PCR amplification with adapter specific primers. The 145–160 bp amplification products were then isolated on a 6% polyacrylamide gel. The concentration of each cDNA library was determined using a Qubit Fluorometer (Invitrogen) and samples were diluted for direct sequencing using the Illumina Hiseq2500 system (San Diego, CA, USA) according to the manufacturer’s protocol.

### 3.5. Basic Data Processing

The raw sequence reads produced by deep sequencing were preprocessed using the FASTX-Toolkit (Available online: http://hannonlab.cshl.edu/fastx_toolkit/). After adapter trimming, low quality reads and reads shorter than 15 nt were removed to obtain clean reads. All clean reads were further annotated and classified by aligning to the pig genome mRAN, miRBase, Rfam and RepBase databases. After alignment to Rfam and Genebank, rRNA, tRNA, snoRNA, snRNA and repeats contamination were discarded and the remaining reads, now considered clean miRNA reads, were analyzed by miRDeep2 to identify known and novel miRNAs in our deep sequencing data [50].

### 3.6. Differential Expression Analysis of miRNAs

To compare miRNA expression data between the LPS and saline groups, expression of each miRNA was normalized to the total number of reads in the sample using the following formula: Normalized expression = (actual miRNA read count/total clean read count) × 1,000,000 [51]. The fold change in miRNA reads was presented as log 2 transformation. Fold-change formula: Fold change = log 2 (LPS group/Saline group). A *p* value less than 0.05 is considered significant.

### 3.7. Cell Culture

C2C12 myoblasts (ATCC, Manassas, VA, USA) were cultured in growth medium (*i.e*., DMEM supplemented with 10% FBS (Gibco, Newcastle, Australia), 100 U/mL penicillin, and streptomycin (Gibco)) at 37 °C under 5% CO_2_. At 80% confluence, myoblast differentiation was induced by incubation for 96 h in differentiation medium (DMEM supplemented with 2% horse serum) to form myotubes. LPS (*E. coli* serotype 026:B6, Sigma Chemical, St. Louis, MO, USA) was dissolved (1000 ng/mL) in saline solution. C2C12 myotubes were treated with 1000 ng/mL LPS for the indicated periods (0, 3, 12 and 24 h). In addition, there were triplicate samples of cells at each time point.

## 4. Conclusions

This is to our knowledge the first study to survey miRNA expression profiles in pig skeletal muscle after a LPS challenge. High throughput sequencing allowed us to characterize inflammatory muscle catabolism-related miRNAs. Importantly, miR-146a-5p and miR-221-5p displayed significant differential expression in LPS-injected pig skeletal muscle and LPS-treated C2C12 myotubes, suggesting the two miRNAs might be involved in acute inflammation processes in skeletal muscle. Further investigations should focus on the potential function of miR-146a-5p and miR-221-5p during inflammatory muscle catabolism, including identification of targets and knock-out experiments at the cellular level.

## Supplementary Materials

Supplementary materials can be found at http://www.mdpi.com/1422-0067/16/09/22438/s1.
