# Supplementary material for: Analysis of MicroRNA Expression Profiles in Weaned Pig Skeletal Muscle after Lipopolysaccharide Challenge
Source: Int J Mol Sci. 2015 Sep 16;16(9):22438–55. doi: 10.3390/ijms160922438 (PMC4613317; doi:10.3390/ijms160922438)
Supplement: Supplementary file 1 [file ijms-16-22438-s001.zip › ijms-97359-Supplemental Information/ijms-97359-Supplementary Information.pdf]

Supplementary Information

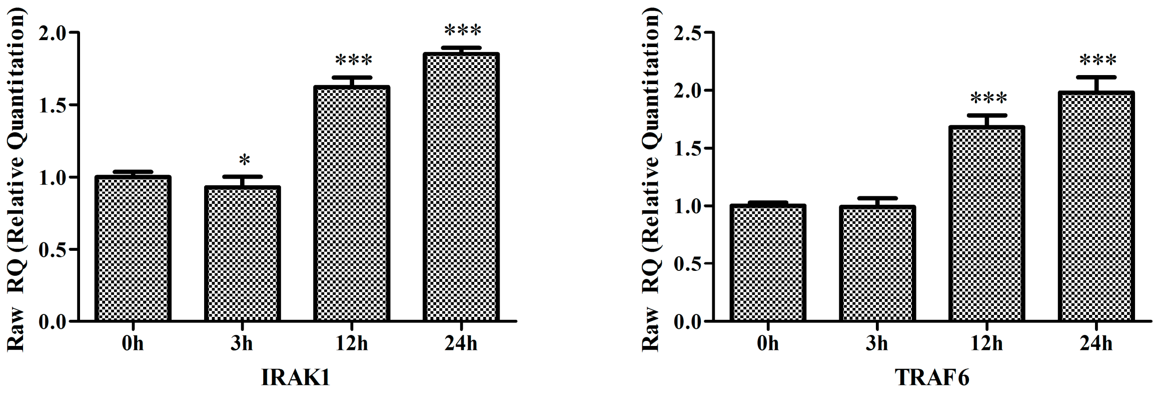

Figure S1. LPS upregulates the expression of IRAK1 and TRAF6 in C2C12 myotubes. C2C12 myotubes were treated with 1000 ng/mL LPS for the indicated periods. Triplicate samples of cells were collected at each time point. RT-qPCR was performed to determine the time-dependent effects of LPS on the expression of IRAK1 and TRAF6. * $p < 0.05$, *** $p < 0.001$ *vs.* 0 h.

Table S4. Specific primers sequences for pigs used for qPCR.

Gene	Forward (5′–3′)	Reverse (5′–3′)
TNF-α	TCCAATGGCAGAGTGGGTATG	AGCTGGTTGTCTTTCAGCTTCAC
TLR4	TCAGTTCTCACCTTCCTCCTG	GTTCATTCCTCACCCAGTCTTC
MyD88	GATGGTAGCGGTTGTCTCTGAT	GTTCATTCCTCACCCAGTCTTC
MAFbx	TCACAGCTCACATCCCTGAG	GACTTGCCGACTCTCTGGAC
MuRF1	ATGGAGAACCTGGAGAAGCA	ACGGTCCATGATCACCTCAT
GAPDH	CGTCCCTGAGACACGATGGT	GCCTTGACTGTGCCGTGGAAT
